# Intraoperative electrophysiological monitoring determines the final electrode position for pallidal stimulation in dystonia patients

**DOI:** 10.3389/fsurg.2023.1206721

**Published:** 2023-05-22

**Authors:** Marek Baláž, Jiří Búřil, Tereza Jurková, Eva Koriťáková, Dušan Hrabovský, Jonáš Kunst, Petra Bártová, Jan Chrastina

**Affiliations:** ^1^ First Department of Neurology, St. Anne’s University Hospital Brno, Faculty of Medicine, Masaryk University, Brno, Czechia; ^2^ Institute of Biostatistics and Analyses, Faculty of Medicine, Masaryk University, Brno, Czechia; ^3^ Department of Neurosurgery, St. Anne’s University Hospital Brno, Faculty of Medicine, Masaryk University, Brno, Czechia; ^4^ Department of Neurology, Faculty Hospital Ostrava, Ostrava, Czechia

**Keywords:** deep brain stimulation, dystonia, globus pallidus internus, microrecordings, stereotaxy

## Abstract

**Background:**

Bilateral deep brain stimulation (DBS) of the globus pallidus internus (GPi) is an effective treatment for refractory dystonia. Neuroradiological target and stimulation electrode trajectory planning with intraoperative microelectrode recordings (MER) and stimulation are used. With improving neuroradiological techniques, the need for MER is in dispute mainly because of the suspected risk of hemorrhage and the impact on clinical post DBS outcome.

**Objective:**

The aim of the study is to compare the preplanned GPi electrode trajectories with final trajectories selected for electrode implantation after electrophysiological monitoring and to discuss the factors potentially responsible for differences between preplanned and final trajectories. Finally, the potential association between the final trajectory selected for electrode implantation and clinical outcome will be analyzed.

**Methods:**

Forty patients underwent bilateral GPi DBS (right-sided implants first) for refractory dystonia. The relationship between preplanned and final trajectories (MicroDrive system) was correlated with patient (gender, age, dystonia type and duration) and surgery characteristics (anesthesia type, postoperative pneumocephalus) and clinical outcome measured using CGI (Clinical Global Impression parameter). The correlation between the preplanned and final trajectories together with CGI was compared between patients 1–20 and 21–40 for the learning curve effect.

**Results:**

The trajectory selected for definitive electrode implantation matched the preplanned trajectory in 72.5% and 70% on the right and left side respectively; 55% had bilateral definitive electrodes implanted along the preplanned trajectories. Statistical analysis did not confirm any of the studied factors as predictor of the difference between the preplanned and final trajectories. Also no association between CGI and final trajectory selected for electrode implantation in the right/left hemisphere has been proven. The percentages of final electrodes implanted along the preplanned trajectory (the correlation between anatomical planning and intraoperative electrophysiology results) did not differ between patients 1–20 and 21–40. Similarly, there were no statistically significant differences in CGI (clinical outcome) between patients 1–20 and 21–40.

**Conclusion:**

The final trajectory selected after electrophysiological study differed from the preplanned trajectory in a significant percentage of patients. No predictor of this difference was identified. The anatomo-electrophysiological difference was not predictive of the clinical outcome (as measured using CGI parameter).

## Introduction

Dystonia is a movement disorder characterized by sustained or intermittent muscle contractions causing abnormal and often repetitive movements or postures or both. Dystonia is often initiated or worsened by voluntary action and associated with overflow muscle activation (1). The pathophysiology includes a loss of inhibition within the sensorimotor circuitry, disrupted sensorimotor integration, and maladaptive homeostatic plasticity of several brain areas (2).

For some cases of dystonia, effective medical treatment is not available. Bilateral pallidal (globus pallidus internus—GPi) deep brain stimulation (DBS) has become a mainstay of therapy for refractory dystonia. Bilateral GPi DBS is an effective treatment for generalized, segmental, and focal isolated (non-neurodegenerative) dystonia (3–5). Tardive dystonia may also respond to GPi DBS (6). In contrast to Parkinson's disease or tremor, therapeutic responses in dystonia are delayed by weeks or months, making it difficult to set the stimulation parameters.

The surgical success and clinical benefits of stimulation depend on several factors, including proper patient selection, absence of fixed contractures, and dystonia type. Correct lead placement is the neurosurgical key point for treatment success (7). For the precise implantation of the stimulation electrode, a combination of neuroradiological target and stereotactic trajectory planning with intraoperative electrophysiology—microelectrode recordings (MER) using parallel simultaneously or sequentially implanted electrodes and intraoperative stimulation is traditionally used (8). However, with the progress in imaging techniques, the need for electrophysiological monitoring for target confirmation is under discussion, mainly because of the prolonged surgical times and potentially increased bleeding risk (9). The first aim of the paper is to analyze the correlation between the preplanned trajectory for GPi stimulation based on a presurgical neuroradiological study and the final trajectory for definitive electrode implantation intraoperatively selected after electrophysiological monitoring. The second aim is to study the factors potentially responsible for the difference between preplanned and final trajectories for definitive electrode implantation: patient (gender, age), disease (dystonia duration and type) (10), and surgical procedure characteristics. The next study aim was to analyze the relationship between increased experience and the correlation between the preplanned and final trajectory for definitive electrode implantation. It has been assumed that as the number of patients operated on by a stable surgical team increases, the correlation between anatomical and electrophysiological targets would improve because of the gained experience (“learning curve effect”).

Finally, an attempt will be made to correlate the selected trajectory for electrode implantation with clinical outcome as (measured using Clinical Global Impression parameter).

## Materials and methods

A group of 40 consecutive patients who underwent bilateral implantation of GPi DBS electrodes using frame-based stereotaxy with intraoperative MER and stimulation for different types of dystonia between 2009 (initiation of GPi DBS program for dystonia patients) and 2020 was studied. All of the patients signed a GPDR consent form according to the European Union regulations pertaining the retention and use of data for clinical research databases. Because the study was a retrospective analysis of prospectively collected clinical data with explicit approval from each included patient not influencing the treatment mode, the approval of the ethics committee was not necessary. No interventions or study-specific procedures were performed. The data were retrospectively collected from a prospectively constructed database of a specialized movement disorder center in a university hospital providing referrals for surgery and postoperative follow-up care. All the surgeries were performed using frame-based techniques (MRI-compatible ceramic frame Leibinger, Zamorano Dujovny stereotactic system Inomed, MicroDrive system Medtronic). Depending on the dystonia severity, the implantations were performed either under general anesthesia (GA) or using neuroleptanalgesia with local anesthesia (NLA).

The imaging protocol included T2 fat-sat in the axial and coronal planes, IR FSE MRI, and postcontrast 3D GE MPR T1 WI. The image sets were merged using stereotactic planning software Praezis Plus, Tatramed Slovakia. The initial coordinates for GPi were defined in reference to the intercommissural (AC-PC) line (19–21 mm laterally from the midline, 2.5 mm in front of the AC-PC midpoint, and 5 mm below the AC-PC line). The coordinates were then modified to the visible contours of GPi on the IR FSE MRI medial to the lamina medullaris interna when visible and in relation to the dorsolateral optic tract, with the lateral and vertical target coordinates matching the dorsolateral border of the optic tract. This point was approximately 2–3 mm anterior to the pallidocapsular border. Attention was also paid to the direction of the trajectory (approximately 60 degrees from the AC-PC line in the sagittal plane and ideally in parallel to the midsagittal plane) avoiding passing the electrodes through the ventricles and blood vessels.

The MicroDrive system (Medtronic, USA) enabled the simultaneous implantation of five parallel microelectrodes. The trajectory defined by the central port of the system is identical to the trajectory based on neuroradiological planning. The remaining 4 ports were marked anterior (2.5 mm anterior to central), lateral (2.5 mm lateral), medial (2.5 mm medial), and posterior (2.5 mm posterior). For microelectrode monitoring during GPi, 4 ports (central, anterior, lateral, and medial) were routinely used. The posterior trajectory was not used because of the expected target proximity to the internal capsule with potential side effect during both intraoperative and chronic stimulation. MER started 10 mm above the anatomical target. Microelectrodes were advanced in 1 mm steps until 5 mm above the anatomical target, and then in 0.5 mm steps. The globus pallidus externus (GPe) could be delineated from the dorsal putamen by an increase in multi-unit firing activity. During further microelectrode advancement, the medial medullary lamina separating the GPe from the GPi with a decrease in electrical activity (thickness 1–2 mm) was reached. The GPi was usually more densely packed with neurons, reflected by more intense multi-unit activity than in GPe. In dystonic patients, bursting and pausing activities were frequently observed in the GPi. The bottom of the GPi was recognized by a sudden decrease in background noise. In GPe and GPi, short and long pauses can occur during MER, with fewer pauses occurring in the GPi, which possesses a more tonic pattern. The GPe cells can also manifest intermittent bursting patterns. Border cells are commonly encountered between the GPe and GPi, inside the GPi (laminae), or on the posterior or ventral border of the GPi. Spontaneous discharge rates of GPi neurons in dystonia were similar to those of GPe neurons; the two nuclei must be distinguished by neuronal discharge patterns rather than rates (8, 11, 12). After MER, stimulation is performed using the electrodes with the best records with the longest and most robust GPi activity recorded (more intense multi-unit activity with bursting and pausing activities) as decided by the electrophysiology monitoring expert in consensus with the operating surgeon. The main aim of intraoperative stimulation was to determine the thresholds for stimulation-induced adverse events (visual, capsular). The type of anesthesia was determined before surgery, following consensus between the anesthesia team, the movement disorder specialist responsible for the intraoperative monitoring, and the neurosurgeon. The use of anesthesia drugs and dosage were tailored individually to the patient's clinical status. In general, the use of benzodiazepines was avoided. Principally, there were two options: total intravenous anesthesia (TIVA—general anesthesia) and analgosedation (AS)—neuroleptanalgesia (NLA). Premedication at night before surgery consists of alprazolam (0.25–0.5 mg tbl.) and on the day of the procedure alprazolam (0.25–0.5 mg tbl.) and paracetamol 1,000 mg tbl, For TIVA, intravenous induction with a combination propofol (1–2 mg/kg; 1% MCT/LCT; Fresenius Kabi GmbH; Germany), sufentanil (10–20 mcg; Sufentanil Torrex; Chiesi Pharmaceuticals GmbH; Austria) and atracurium (0.5 mg/kg; Atracurium; AS Kalcex, Latvia) was followed by anesthesia maintenance with propofol 2% (6–12 mg/kg/h) and additional sufentanil as needed. For periprocedural AS midazolam and sufentanil (as needed) were used.

The right-sided electrode was implanted first in all patients. After the implantation of both electrodes and wound closure, stereotactic CT was performed to verify electrode positions and exclude complications. The pulse generator and connecting cable implantations were performed after postoperative CT study completion and analysis. The MicroDrive ports selected for definitive electrode implantation on both right and left side were recorded in the surgical report.

The factors characterizing the patient (gender and age), dystonia (type and duration) and surgical aspects (thickness of intracranial air on postoperative CT potentially indicating the degree of brain shift and any history of previous lesional intracranial surgery) were extracted from hospital and movement center databases. To evaluate the impact of the team experience on the correlation between the planned and finally selected trajectory for the definitive electrode implantation, the studied group of 40 patients was divided into two subgroups: Group 1 (Patients 1–20) and Group 2 (Patients 21–40). The percentages of final electrodes implanted as related to the MicroDrive ports was compared between both groups for both the right and left sides separately.

The final clinical outcome was (due to the fact, that the studied group included four dystonia types) defined using the modified Clinical Global Impression scale. Compared to the clinical status before surgery, this patient's condition was: 1 = very much improved since the initiation of treatment; 2 = much improved; 3 = minimally improved; 4 = no change from baseline (the initiation of treatment); 5 = minimally worse; 6 = much worse; 7 = very much worse since the initiation of treatment (13).

For the descriptive statistics, continuous variables were represented by median, 25th percentile, 75th percentile, and minimum and maximum values, or by median, minimum/maximum values, mean and standard deviation (SD) for the learning curve study. Binary (qualitative) variables were represented by absolute numbers and percentages. McNemar's test was used to determine whether there was a statistically significant difference in the MicroDrive ports used for definitive electrode implantation between the right and left hemispheres. Wilcoxon paired test was used to test the difference in the thickness of postoperative air between the right and left hemispheres. The study of the potential predictors of the final electrode position was performed for the right-brain and left-brain hemispheres separately. All predictors carrying electrode position information were descriptively summarized and compared using Fisher's exact test. A binary logistic regression model was used to model the final electrode position for the right or left hemisphere. In addition to the odds ratio (OR), the tables for one-dimensional models also show a 95% confidence interval (CI) and the corresponding *p* values. The software R 3.6.0 and software IBM SPSS Statistics 26 were used. All tests were performed at a significance level of *p* = .05.

## Results

The clinical characteristics of the included patients are summarized in Table 1.

**Table 1:** Patient characteristics.

Gender	*n* (%)
Male	26 (65.0%)
Female	14 (35.0%)
Dystonia type	*n* (%)
Focal dystonia	13 (32.5%)
Generalised dystonia	17 (42.5%)
Multifocal dystonia	2 (5.0%)
Segmental dystonia	8 (20.0%)
Age at surgery	[years]
Median (25th percentile–75th percentile)	43.0 (31.8–51.3)
Minimum–maximum	10.0–69.0
Duration of dystonia	[years]
Median (25th percentile–75th percentile)	11.5 (5.0–26.0)
Minimum–maximum	1.0–38.0

The distribution of the MicroDrive ports selected for final electrode implantation for both right and left brain hemispheres is presented in Table 2.

**Table 2:** Final electrode position for right- and left-brain hemisphere as related to MicroDrive ports.

Definitive electrode position	Right hemisphere		Left hemisphere	
	*n*	%	*n*	%
Central	29	72.5%	28	70.0%
Anterior	5	12.5%	4	10.0%
Lateral	1	2.5%	1	2.5%
Medial	5	12.5%	7	17.5%

No hemorrhagic complication requiring surgical treatment was observed. In one patient, two small asymptomatic intraparenchymal hematomas (less than 8 mm) along the right GPi electrode in the white matter were found on postoperative CT. In another two patients, subarachnoid hemorrhage was found in the vicinity of electrode entry into the brain (also not requiring surgery). The incidence of symptomatic hemorrhagic complications was 0%, and the incidence of asymptomatic bleedings detectable on postoperative CT was 3.75% per electrode.

The majority of the electrodes were implanted in the anatomic trajectory—the central port of the MicroDrive system—72.5% on the right side and 70.0% on the left side, followed by medial and anterior positions. In 22 patients (55%), central electrodes were bilaterally implanted. A trajectory other than the anatomical (central port of the MicroDrive system) in both hemispheres was used in 5 (12.5%) patients. In all patients the selected trajectory for definitive electrode implantation reflected the longest and the most robust MER even after intraoperative stimulation. In other words no adverse stimulation induced effects requiring trajectory change from that with the best MER were observed. There was no statistically significant difference among the frequencies of trajectories between the right and left hemispheres (McNemar's test *p* = .998).

In 40% of patients, GA (TIVA) was required; local anesthesia with sedation was sufficient in 60% of patients. The position of the definitive electrodes in the right and left hemispheres for GA and AS (NLA) is summarized in Table 3. There were no adverse events associated with the type of anesthesia selected, with one exception of poorly controllable restlessness with fixation screw breakage immediately after implantation of the left (second implanted) final GPi electrode requiring quick surgery completion, stereotactic frame removal, and a CT scan that excluded surgical complications and proved the correct position of the final electrode.

**Table 3:** Final electrode position for right- and left-brain hemisphere in general anaesthesia (GA) and neuroleptanalgesia (NLA).

Electrode position MicroDrive ports	GA (*n* = 16)		NLA (*n* = 24)	
	R hemisphere	L hemisphere	R hemisphere	L hemisphere
	*n* (%)	*n* (%)	*n* (%)	*n* (%)
Central	12 (75.0%)	11 (68.8%)	17 (70.8%)	17 (70.8%)
Anterior	3 (18.8%)	2 (12.5%)	2 (8.3%)	2 (8.3%)
Lateral	0 (0.0%)	0 (0.0%)	1 (4.2%)	1 (4.2%)
Medial	1 (6.3%)	3 (18.8%)	4 (16.7%)	4 (16.7%)

The maximum thickness of frontal pneumocephalus in mm was measured on the postoperative CT scans (Table 4).

**Table 4:** Intracranial pneumocephalus thickness.

Intracranial air on the right hemisphere	[mm]
Median (25th percentile–75th percentile)	5.0 (1.0–7.0)
Minimum–maximum	0.0–20.0
Intracranial air on the left hemisphere	[mm]
Median (25th percentile–75th percentile)	5.0 (1.0–7.0)
Minimum–maximum	0.0–17.0

No difference in the thickness of frontal pneumocephalus between the right and left side was found (*p *= .258). Table 5 summarizes the potential predictors of the difference between the preplanned and final electrode trajectories for the right and left hemispheres. No statistically significant difference (contingency tables) was demonstrated between patients with the final electrode implanted along the preplanned trajectory (central port of the MicroDrive system) and patients with the final electrode in other positions for any factors. Similarly, there is no association between CGI (clinical outcome) and final trajectory selected for electrode implantation (MicroDrive system) in the right/left hemisphere. From the point of anatomo-electrophysiological correlation there was no statistically significant difference in the frequency of CGI categories between the central (anatomical) trajectories or trajectories modified after MER and intraoperative stimulation.

**Table 5:** Location of final electrodes on the right and left hemisphere.

Variable	Central final electrode position (MicroDrive system) right hemisphere		Central final electrode position (MicroDrive system) left hemisphere	
	Yes	No	Yes	No
	29 (72.5%)	11 (27.5%)	28 (70.0%)	12 (30.0%)
Gender				
Female	10 (34.5%)	4 (36.4%)	9 (32.1%)	5 (41.7%)
Male	19 (65.5%)	7 (63.6%)	19 (67.9%)	7 (58.3%)
* *	*p* value > .999		*p* value = .720	
Age category [years]				
≤20	3 (10.3%)	0 (0.0%)	3 (10.7%)	0 (0.0%)
21–40	8 (27.6%)	6 (54.5%)	9 (32.1%)	5 (41.7%)
41–60	14 (48.3%)	4 (36.4%)	14 (50.0%)	4 (33.3%)
>60	4 (13.8%)	1 (9.1%)	2 (7.1%)	3 (25.0%)
	*p* value = .476		*p* value = .290	
Duration of dystonia [years]				
≤10	12 (41.4%)	5 (45.5%)	12 (42.9%)	5 (41.7%)
11–20	8 (27.6%)	3 (27.3%)	9 (32.1%)	2 (16.7%)
21–30	5 (17.2%)	3 (27.3%)	5 (17.9%)	3 (25.0%)
>30	4 (13.8%)	0 (0.0%)	2 (7.1%)	2 (16.7%)
	*p* value = .723		*p* value = .674	
Previous surgery				
Yes	2 (6.9%)	1 (9.1%)	2 (7.1%)	1 (8.3%)
No	27 (93.1%)	10 (90.9%)	26 (92.9%)	11 (91.7%)
	*p* value > .999		*p* value > .999	
Dystonia type				
Focal	10 (34.5%)	3 (27.3%)	9 (32.1%)	4 (33.3%)
Generalised	14 (48.3%)	3 (27.3%)	12 (42.9%)	5 (41.7%)
Multifocal	1 (3.4%)	1 (9.1%)	1 (3.6%)	1 (8.3%)
Segmental	4 (13.8%)	4 (36.4%)	6 (21.4%)	2 (16.7%)
	*p* value = .279		*p* value > .999	
Intracranial air in the right hemisphere				
0	5 (17.2%)	2 (18.2%)	5 (17.9%)	2 (16.7%)
1–5	10 (34.5%)	5 (45.5%)	11 (39.3%)	4 (33.3%)
6–10	9 (31.0%)	4 (36.4%)	8 (28.6%)	5 (41.7%)
>10	5 (17.2%)	0 (0.0%)	4 (14.3%)	1 (8.3%)
	*p* value = .594		*p* value = .923	
Intracranial air in the left hemisphere				
0	6 (20.7%)	2 (18.2%)	5 (17.9%)	3 (25.0%)
1–5	11 (37.9%)	3 (27.3%)	10 (35.7%)	4 (33.3%)
6–10	9 (31.0%)	6 (54.5%)	11 (39.3%)	4 (33.3%)
>10	3 (10.3%)	0 (0.0%)	2 (7.1%)	1 (8.3%)
	*p* value = .620		*p* value = .955	
Anaesthesia				
General anaesthesia	12 (41.4%)	4 (36.4%)	11 (39.3%)	5 (41.7%)
Neuroleptanalgesia	17 (58.6%)	7 (63.6%)	17 (60.7%)	7 (58.3%)
	*p* value > .999		*p* value > .999	
CGI				
1	3 (10.3%)	3 (27.3%)	2 (7.1%)	4 (33.3%)
2	16 (55.2%)	4 (36.4%)	16 (57.1%)	4 (33.3%)
3	6 (20.7%)	2 (18.2%)	6 (21.4%)	2 (16.7%)
4	3 (10.3%)	2 (18.2%)	3 (10.7%)	2 (16.7%)
5	1 (3.4%)	0 (0.0%)	1 (3.6%)	0 (0.0%)
	*p* value = .612		*p* value = .230	

A one-dimensional binary logistic regression model confirmed the data from the contingency tables. All *p* values were greater than the significance level of *p* = .05. No statistically significant effect was proven for any of the studied variables on the final electrode position as defined by the MicroDrive system ports for each hemisphere (Table 6).

**Table 6:** One-dimensional binary logistic regression study for analysing factors with potential importance for the final electrode position and CGI categories.

Predictor	Other than central electrode position (R)			Other than central electrode position (L)		
	OR	95% CI	*p* value	OR	95% CI	*p* value
Gender (M/F)	0.921	(0.217; 3.917)	.911	0.663	(0.164; 2.676)	.564
Age	0.986	(0.939; 1.034)	.560	1.026	(0.977; 1.078)	.301
Duration of dystonia	0.996	(0.936; 1.060)	.897	1.030	(0.970; 1.093)	.332
Previous surgery (Y/N)	1.350	(0.110; 16.574)	.815	1.182	(0.097; 14.425)	.896
Dystonia type						
Generalized/focal	0.714	(0.119; 4.297)	.713	0.938	(0.194; 4.522)	.936
Multifocal/focal	3.333	(0.157; 70.910)	.440	2.250	(0.111; 45.725)	.598
Segmental/focal	3.333	(0.502; 22.143)	.213	0.750	(0.103; 5.470)	.777
Intracranial air (R)	0.876	(0.730; 1.051)	.154	0.999	(0.867; 1.153)	.994
Intracranial air (L)	1.012	(0.885; 1.193)	.725	0.998	(0.860; 1.157)	.976
Anaesthesia (NLA/GA)	1.235	(0.295; 5.181)	.773	0.906	(0.229; 3.585)	.888
CGI						
1/4	1.500	(0.136; 16.543)	.741	3.000	(0.255; 35.336)	.383
2/4	0.375	(0.046; 3.056)	.360	0.375	(0.046; 3.056)	.360
3/4	0.500	(0.045; 5.514)	.571	0.500	(0.045; 5.514)	.571
5/4	0.000	(0.000 ; Inf)	.995	0.000	(0.000 ; Inf)	.995

For the CGI variable, we have selected category 4 as the reference category. There was no statistically significant difference between the reference category and the other categories in the final electrode placement on the left or right hemisphere.

Table 7 shows the distribution of right-sided GPi electrodes as related to the MicroDrive system ports for Group 1 (Patients 1–20) and Group 2 (Patients 21–40).

**Table 7:** Distribution of electrodes in the right hemisphere in group 1 and group 2.

	Patients 1–20	Patients 21–40
	*N* (%)	*N* (%)
Definitive electrode trajectory in the right hemisphere		
Anterior	2 (10.0%)	3 (15.0%)
Central	14 (70.0%)	15 (75.0%)
Lateral	1 (5.0%)	0 (0.0%)
Medial	3 (15.0%)	2 (10.0%)

The percentage of central electrodes is slightly higher in Group 2 (70% Group 1, 75% Group 2). However, the *p* value of Fisher's exact test is >.999, therefore the statistically significant difference in the distribution of electrodes between both groups has not been confirmed.

Table 8 shows the distribution of left-sided GPi electrodes (second implanted) as related to the MicroDrive system ports for Group 1 and 2.

**Table 8:** Distribution of electrodes in the left hemisphere in group 1 and group 2.

	Patients 1–20	Patients 21–40
	*N* (%)	*N* (%)
Definitive electrode trajectory in the left hemisphere		
Anterior	3 (15.0%)	1 (5.0%)
Central	13 (65.0%)	15 (75.0%)
Lateral	1 (5.0%)	0 (0.0%)
Medial	3 (15.0%)	4 (20.0%)

Although the percentage of central electrodes is higher in Group 2 (65% Group 1, 75% Group 2), the *p* value of Fisher's exact test is.682. Therefore, the statistically significant difference in the distribution of electrodes between both groups has not been confirmed for the left hemisphere.

The summary and comparison of demographic, clinical, and surgical characteristics of both groups are summarized in Table 9 (categorical variables) and Table 10 (continuous variables). For categorical variables, Fisher's test did not prove a statistically significant difference between Groups 1 and 2 for any of the studied parameters. It is also important to note that there were no statistically significant differences in CGI between patients 1–20 and 21–40.

**Table 9:** Statistical analysis of categorical variables in group 1 and group 2.

	Patients 1–20	Patients 21–40	Fisher's exact test
Gender			
Female	8 (40.0%)	6 (30.0%)	.741
Male	12 (60.0%)	14 (70.0%)	
Anesthesia			
Central anesthesia	5 (25.0%)	11 (55.0%)	.105
Neuroleptanesthesia	15 (75.0%)	9 (45.0%)	
Previous surgery			
Yes	3 (15.0%)	0 (0.0%)	.231
No	17 (85.0%)	20 (100.0%)	
Dystonia type			
Focal	6 (30.0%)	7 (35.0%)	.987
Generalized	9 (45.0%)	8 (40.0%)	
Multifocal	1 (5.0%)	1 (5.0%)	
Segmental	4 (20.0%)	4 (20.0%)	
CGI			
1	3 (15.0%)	3 (15.0%)	.571
2	10 (50.0%)	10 (50.0%)	
3	3 (15.0%)	5 (25.0%)	
4	4 (20.0%)	1 (5.0%)	
5	0 (0.0%)	1 (5.0%)	

**Table 10:** Statistical analysis of continuous variables in group 1 and group 2.

	Patients 1–20	Patients 21–40	Mann-Whitney test
Age at the time of implantation			
Mean (SD)	48 (13)	37 (14)	.041
Median	47	38	
Min–max	30–69	10–58	
Duration of dystonia [years]			
Mean (SD)	17 (11)	13 (11)	.151
Median	15	11	
Min–max	3–36	1–38	
Intracranial air in the right hemisphere			
Mean (SD)	5 (6)	6 (3)	.165
Median	2	6	
Min–max	0–20	0–13	
Intracranial air in the left hemisphere			
Mean (SD)	5 (6)	5 (3)	.235
Median	1	6	
Min–max	0–17	0–10	

For continuous variables according to the results of the Mann-Whitney test, the patients in Group 2 were significantly younger than in Group 1 (*p* = .041).

## Discussion

### General remarks

Bilateral GPi stimulation is currently the preferred treatment mode in the majority of patients with refractory dystonia indicated for DBS. A multicentric randomized study of bilateral GPi stimulation for primarily generalized or segmental dystonia confirmed a significantly better improvement at the 3-month follow-up visit in the surgical group (39.4%) than in controls (4.3%) (3). The target and electrode trajectory is traditionally determined using a combination of neuroradiological planning, intraoperative electrophysiological monitoring (MER), and response to the test stimulation. The main points in the discussion about the role of intraoperative electrophysiological monitoring are the potentially higher risk of hemorrhagic complications, the difference between the preplanned and final trajectory for definitive electrode implantation and the responsible factors, and the association between clinical outcome and the use of intraoperative electrophysiology.

### Hemorrhagic complications

The problems with the potential association between MER and hemorrhagic complications have been extensively studied. Sansur et al. reported a higher incidence of hemorrhage in patients with MER (3.40%) than in patients without MER (0.75%), but the reported difference was not statistically significant. The risk of symptomatic hemorrhage was 1.2% and the risk of permanent neurological deficit was 0.7%. Parkinson's disease was reported as one of the risk factors of postoperative hemorrhage (14). When considering the topic of the paper it is important to note that according to Binder et al., the incidence of hemorrhagic complications is higher in pallidal stimulation (6.7% from all electrodes) than in subthalamic stimulation (2.5%) (15). In an extensive metanalysis combined with their own series of 214 patients, Zrinzo et al. reported that the incidence of symptomatic hemorrhage and hemorrhage leading to permanent deficit in studies adopting an image-guided and image-verified approach without MER was significantly lower than that reported with other techniques. In the series of 214 patients operated on by the authors, the incidence of hemorrhages in dystonic patients was 0%. DBS for dystonia was associated with lower surgical risks than DBS for Parkinson's disease (9). On the other side Bezchlibnyk et al. report the rate of serious complications at 6.9% for MER-guided DBS lead placement and 9.4% for intraoperative MRI-guided DBS lead placement (16). In our study, the incidence of asymptomatic small hemorrhages not affecting deep brain structures was 3.75% (per electrode), and the incidence of symptomatic hemorrhages was 0%, which matches the preceding data.

### The difference between the preplanned and final target for electrode implantation

The main fact supporting the use of MER is the difference between the preplanned (anatomical) trajectory and final trajectory for the definitive electrode implantation (MicroDrive system port) selected after intraoperative monitoring. In our study, the final electrode was implanted along the preplanned (anatomical—central MicroDrive port) trajectory in 72.5% of patients on the first implanted right side and in 70.0% of patients on the left side, with medial (right 12.5%, left 17.5%) and anterior (right 12.5%, left 10%) trajectories being less frequently used. In only 55% of the patients both electrodes were implanted along the central trajectory, and in 12.5% of patients none of the final electrodes matched the anatomical trajectory. In a paper by Bour et al., the percentage of central trajectories of GPi electrodes was 64% on the first implanted left side and 50% on the right side, followed by lateral (right side 34%, left side 9%) and medial trajectories (right side 8%, left side 18%). Posterior and anterior trajectories were used rarely. The trend towards a less frequent choice for the central channel on the right side (second implantation) was not significant (12).

In a paper by Pinsker et al., definitive electrodes were implanted along the central trajectory in 64% of the hemispheres. The medial trajectory was the second most frequent (20%), followed by the anterior trajectory (9%). Posterior trajectories were used rarely (3.5% each). The percentage of patients with both electrodes implanted in the central trajectory (47.6%) and the percentage of patients with both trajectories changed after MER and stimulation (14.3%) is comparable with our results. Unfortunately, the authors did not present the results for each hemisphere separately (17).

In our group, the posterior electrode trajectory was not used because of the relationship of the electrode trajectory to the internal capsule. Although the potential proximity of the medial MicroDrive port trajectory to the internal capsule should be also considered, in none of our patients with definitive electrode implanted in the medial trajectory based on the most robust MER data, no stimulation related capsular adverse events were observed. The avoidance of the posterior electrode was supported by a study by Bour et al. Although MER activity was highest at the posterior electrode in 20% of the cases, this channel was selected only for permanent stimulation only in two patients due to capsular side effects (12).

The potential causes of the reported anatomo-electrophysiological discrepancy may be intraoperative brain shift, the non-correlating results of different targeting techniques, spatial distortion of magnetic resonance images, anatomical variability of the target structures, interpretations of radiological and electrophysiological data, and possible technical inaccuracies during surgery (18–20).

### The role of brain shift

According to Miyagi et al., intracranial air entry after dural opening for the implantation of the first electrode results in contralateral and dorsal (posterior) brain shift. After durotomy for the second electrode implantation, equilibrium is established in a mediolateral direction, but dorsal shift persists (21). The less marked correlation between MRI planning and MER on the second operative side was attributed to intraoperative cerebrospinal fluid loss, and subsequent subdural air influx potentially causing posterior brain displacement more pronounced on the second side operated on (12, 22). However, a study by Ivan et al. investigating brain shift in various regions of the brain during DBS electrode implantation throughout the procedure with high-field interventional MRI did not confirm this simplifying assumption. According to the authors, the degree of brain shift and its direction were unpredictable. Although shifts in the pallidum and subthalamic region ipsilateral to the burr hole averaged 0.6 mm, which is not significant considering the fact that the diameter of the DBS electrode is 1.2 mm, 9% of patients had over 2 mm of shift in deep brain structures (23). The data from our study do not confirm a significantly lower percentage of central electrodes on the left side (second operated on). Moreover, if the cause of the difference between anatomical and final trajectory is a posterior brain shift, then a lower percentage of anterior electrodes could be expected on the left side. The percentage of anterior electrodes in our group was nearly identical for right and left hemispheres (right 12.5%, left 10%).

### Other factors

Our results did not confirm the significance of age, disease duration, dystonia type, or previous surgeries for the difference between anatomical and final trajectory. Theoretically, in older patients with longer disease duration, structural changes of the target structures can be expected that could require trajectory modification. Similarly, cerebral atrophy with larger subarachnoid spaces create the potential for more intraoperative cerebrospinal fluid loss with greater potential for brain shift affecting target structures. However, the statistical analysis did not confirm this assumption. In older patients, smaller GPi volume due to brain atrophy was considered as one of the factors potentially influencing both the neuroradiological target planning and intraoperative microrecordings. This hypothesis is partially supported by a paper by Vasques et al. The authors analyzed the impact of GPi volume on the results of deep brain stimulation (DBS) by comparing highly and less improved patients with primary dystonodyskinetic syndromes. The authors showed that the mean volume of the right and left GPi in patients showing less response to DBS was significantly smaller than the GPi volume of patients who responded well (24).

When implanting GPi electrodes after lesional surgery (thalamotomies in all our patients), the anatomy may be distorted by the surgical lesion, and less accurate anatomo-electrophysiological correlations therefore can be expected. However only three patients underwent bilateral GPi implantation after unilateral thalamotomy. Although anatomical (central MicroDrive) trajectories were used in two of them, this small number precludes any definitive considerations.

### Local versus general anesthesia

Heavy sedation or general anesthesia is sometimes needed for patients who are unable to tolerate the procedure awake because of severe dystonic symptoms, but also due to psychological distress, other forms of discomfort, or mental disability. The effect of anesthetic drugs on MER is controversial but likely depends on the type and dose of a particular anesthetic agent, underlying disease severity, and surgical target. Several studies have shown that anesthetic drugs decrease neuronal firing in patients with dystonia (25, 26).

The depressant influence of sedative agents on neuronal activity can partly be explained by the large amount of gamma-aminobutyric acid (GABA) input to the target nuclei. It has been shown that enhancement of GABAergic input alters the level and pattern of firing activity of pallidal neurons in normal and pathological conditions (27). General anesthesia may not only influence the quality of MER, but also preclude the identification of some adverse events during stimulation (e.g., flashing when stimulating in the proximity of the optic tract). Anesthetic agents such as benzodiazepines and propofol potentiate the inhibitory actions of GABA with a major effect on single-cell activity. The effect is dose dependent; however, the general consensus is to avoid the use of benzodiazepines in DBS surgery, which was also respected in our study group (28). Generally speaking, in DBS surgery, remifentanil is often combined with propofol or dexmedetomidine. Although a retrospective review by Venkatraghavan et al. suggests a difference in spontaneous and evoked neuronal discharges with MER performed under general anesthesia compared with no sedation (29), it was possible to obtain valid recordings in all included patients enabling safe implantation of the definitive electrode. In our study the percentage of central trajectories for each hemisphere did not differ between the patients operated on under general anesthesia and those with local/neuroleptanalgesia.

### Learning curve

The impact of the factors responsible for the anatomo-electrophysiological disturbance—unrecognized anatomical variability of the target structures, interpretations of radiological and electrophysiological data, possible technical inaccuracies during surgery, and shortcomings of stereotactic atlases used for indirect target planning (30) can be influenced to some degree by the experience of the surgical team; therefore, the learning curve must also be considered, not only in terms of surgical complications and adverse events. Pallavaram et al. studied the intersurgeon variability in the principal task of stereotactic planning—the manual selection of the anterior and posterior commissures together with the impact of this variability on the localization of targets like the subthalamic nucleus, ventralis intermedius nucleus, and GPi. These data also show that even for experienced neurosurgeons, variations in selecting the AC and the PC point result in substantial variations at the target points. These variations are larger when residents/fellows are included in the data set (31). Hrabovsky et al. studied the learning curve effect in subthalamic electrode implantation implanted by a stable surgical team. They compared the percentage of electrodes implanted in the preplanned (central MicroDrive port) trajectory in patients 1–50 and 51–100 for the right (first implanted) and left hemispheres. The results confirmed a higher percentage of central electrodes on the first side implanted in patients 1–50 (right 52%, left 38%). The percentages of central electrodes in patients 51–100 substantially increased on the right (76%) and left sides (78%), thereby confirming the learning curve effect (32). However, this learning curve effect in GPi patients has not been confirmed. One potential explanation is the smaller sample size. Another explanation may be the larger volume of the target structure. The GPi is larger (400–500 mm^3^) than the subthalamic nucleus (110–150 mm^3^). However, the best target to treat dystonic symptoms is the lateral posteroventral GPi (sensorimotor territory) with the effective volume of tissue activated being 153 mm^3^ (24, 33).

Atlases using multimodal MRI, histology, and structural connectivity have defined the primary motor, sensory, and sensorimotor regions of the GPi with somatotopic organization (upper extremities located ventral and lateral to the lower limb area; orofacial movements located further ventral to the upper extremities movements). The possibility of intraoperative identification of the GPi somatotopy in individual patients using MER and stimulation techniques has the potential to enhance the efficacy of GPi DBS, especially in focal and segmental dystonias, through the more precise targeting of the stimulation electrode and directing of the stimulating current (12, 29, 34).

### Further considerations

The study did not prove the correlation between the selected trajectory for definitive electrode implantation (central—anatomical versus not central—modified after intraoperative microrecording). This problem is an interesting topic for further study with a larger pool of patients, mainly because of the need to differentiate between the various dystonia types with differing prognoses of GPi stimulation. The impact of MER on clinical outcomes is not clear even in the widely studied subthalamic stimulation for Parkinson's disease (35–38). The published groups of patients with GPi DBS implanted for dystonia are much less extensive than the published studies of Parkinson's disease patients, with some exceptions, such as the study by Maldonado et al. with 478 electrodes implanted, 426 for dystonic-dyskinetic syndromes and 52 for Parkinson's disease (39). To our knowledge, the only paper dealing with the relation of clinical outcomes to MER results in dystonic patients was published by Pinsker et al. The median improvement in dystonic patients after bilateral GPi stimulation did not differ significantly between the patients with final electrode implanted along the central trajectory and patients with the final trajectory modified after intraoperative electrophysiological study (17). Although Pinsker et al. used different criteria for outcome evaluation our results (no significant difference in CGI between patients with final electrode implanted along central—anatomical trajectory and final electrode implanted along non central trajectory selected after electrophysiological monitoring) match their data. Two maybe simplifying conclusions can be drawn from this result. Should the clinical outcomes be better in patients with electrodes implanted along non central trajectory, this situation indicates problem with the anatomical planning. Similarly, if the clinical outcomes are better in patients with electrodes implanted along the central trajectory, this situation suggests a problem with intraoperative electrophysiology (not only because of interpretation). However, the question about the role of intraoperative electrophysiological monitoring including MER not only in dystonic patients can be answered only by a well-designed study with a large numbers of matched patients operated on by image-guided technique and MER experts, as proposed by Bakay et al. (40).

## Conclusion

The study of the intraoperative correlation between the anatomy-based preplanned trajectory and final trajectory defined by the MicroDrive port selected for final electrode implantation study addresses one of the most important aspects of the complex problem of DBS treatment in dystonia patients. Despite meticulous presurgical planning, the final trajectory of the MicroDrive system selected for GPi electrode implantation differed from the preplanned (central MicroDrive port) trajectory based on neuroradiological data in at least one brain hemisphere in 45% of patients. The percentage of electrodes implanted along the anatomical trajectory did not differ between the first implanted right-brain and left-brain hemispheres. A detailed statistical study did not confirm any of the investigated factors (gender, age, disease duration, dystonia type, need for general anesthesia, amount of intracranial air, electrode displacement in the opposite hemisphere, and previous surgery) as a possible predictor of the difference between neuroradiological and final trajectory for each hemisphere separately. Regarding the impact of the final trajectory selected for definitive electrode implantation no significant difference in clinical outcome (CGI) between patients with final electrode implanted along central—anatomical trajectory and final electrode implanted along non central trajectory selected after electrophysiological monitoring. The effect of learning curve both on the anatomo-electrophysiological correlation and the clinical outcome (CGI) has not been shown. Despite the advances in neuroradiological techniques and the absence of a study directly comparing clinical outcomes of intraoperative electrophysiology and non-intraoperative electrophysiology patients the difference between the preplanned and final electrode positions supports the continued use of intraoperative monitoring in GPi DBS electrode implantation.

## Data Availability

The raw data supporting the conclusions of this article will be made available by the authors, without undue reservation.
